# Anti-bacterial and Anti-biofilm Evaluation of Thiazolopyrimidinone Derivatives Targeting the Histidine Kinase YycG Protein of *Staphylococcus epidermidis*

**DOI:** 10.3389/fmicb.2017.00549

**Published:** 2017-03-31

**Authors:** Zhihui Lv, Dan Zhao, Jun Chang, Huayong Liu, Xiaofei Wang, Jinxin Zheng, Renzheng Huang, Zhiwei Lin, Yongpeng Shang, Lina Ye, Yang Wu, Shiqing Han, Di Qu

**Affiliations:** ^1^Key Laboratory of Medical Molecular Virology of Ministry of Education and Ministry of Public Health, Institute of Medical Microbiology and Institutes of Biomedical Sciences, Shanghai Medical School of Fudan UniversityShanghai, China; ^2^College of Biotechnology and Pharmaceutical Engineering, Nanjing Tech UniversityNanjing, China; ^3^Department of Natural Products Chemistry, School of Pharmacy, Fudan UniversityShanghai, China; ^4^Department of Infectious Diseases and Shenzhen Key Lab for Endogenous Infection, Shenzhen Nanshan Hospital, Shenzhen UniversityShenzhen, China; ^5^Department of Gastroenterology, Zhongshan Hospital of Fudan UniversityShanghai, China

**Keywords:** *Staphylococcus epidermidis*, histidine kinase YycG, minimal inhibitory concentration, minimal bactericidal concentration, anti-biofilm activity

## Abstract

Staphylococcus epidermidis is one of the most important opportunistic pathogens in nosocomial infections. The main pathogenicity associated with *S. epidermidis* involves the formation of biofilms on implanted medical devices, biofilms dramatically decrease the efficacy of conventional antibiotics and the host immune system. This emphasizes the urgent need for designing novel anti-staphylococcal biofilm agents. Based on the findings that compound 5, targeting the histidine kinase domain of *S. epidermidis* YycG, possessed bactericidal activity against staphylococci, 39 derivatives of compound 5 with intact thiazolopyrimidinone core structures were newly designed, 7 derivatives were further screened to explore their anti-bacterial and anti-biofilm activities. The seven derivatives strongly inhibited the growth of *S. epidermidis* and *Staphylococcus aureus* in the minimal inhibitory concentration range of 1.56–6.25 μM. All the derivatives reduced the proportion of viable cells in mature biofilms. They all displayed low cytotoxicity on mammalian cells and were not hemolytic to human erythrocytes. The biofilm inhibition activities of four derivatives (H5-32, H5-33, H5-34, and H5-35) were further investigated under shearing forces, they all led to significant decreases in the biofilm formation of *S. epidermidis*. These results were suggestive that the seven derivatives of compound 5 have the potential to be developed into agents for eradicating biofilm-associated infections.

## Introduction

*Staphylococcus epidermidis* is one of the most important opportunistic pathogens in nosocomial infections, in particular, it is the leading cause of biofilm infections related to implanted medical devices (Otto, 2009). Once a biofilm has been established on these devices, it is difficult to eradicate because the bacteria in a biofilm are protected from attack by the host immune system and are generally much more resistant to antibiotics or biocides than their planktonic counterparts (Høiby et al., 2011). A possible mechanism underlying this resistance is that biofilm-encased bacteria might restrict the diffusion of antibiotics due to interactions with the exopolysaccharide matrix (Lewis, 2001). Currently, the effective treatment for such biofilm infections is to remove or replace the implanted devices, resulting in substantial healthcare costs (Vuong and Otto, 2002). Biofilms are also associated with the emergence and spread of antimicrobial resistance (Römling and Balsalobre, 2012; Savage et al., 2013). In addition, with the excessive use of antibiotics in hospitals, there have been more and more reports of multi-drug resistance in staphylococci, resulting in most of the available agents having a limited efficacy in treating serious infections caused by staphylococci, such as prosthetic valve endocarditis and blood infections (Raad et al., 1998; Ventola, 2015). New strategies for combating biofilm infections, therefore, have become challenging and are attracting considerable scientific attention (Sadekuzzaman et al., 2015). In the past few decades, the development of bacterial genomics, bioinformatics, and gene manipulation have brought about new opportunities for discovering many novel protein targets for anti-bacterial agents (Moir et al., 1999; Silver, 2011).

Two-component systems (TCSs) are important regulators in most bacteria for cell adaption to environmental conditions (Stock et al., 2000; Bijlsma and Groisman, 2003). A TCS usually consists of a histidine kinase (HK) and a response regulator (Stock et al., 2000). TCSs have no human homologs or structurally similar proteins (Bem et al., 2015); thus, they have attracted increasing attention as potential anti-bacterial targets (Macielag and Goldschmidt, 2000). Among TCSs, YycG/YycF (also known as WalK/WalR) is essential in low G+C Gram-positive bacteria, such as *Bacillus subtilis, Enterococcus faecalis, Staphylococcus aureus*, and *Streptococcus pneumoniae* (Fabret and Hoch, 1998; Martin et al., 1999; Throup et al., 2000; Hancock and Perego, 2002). It has been shown that inhibitors acting directly against YycG or YycF (WalR) have bactericidal effects and are active against an array of clinically important Gram-positive pathogens (Watanabe et al., 2003; Gotoh et al., 2010; Okada et al., 2010). Using a structure-based virtual screening approach, we have found two leading compounds (compound 2 and compound 5) targeting YycG that possess not only bactericidal but also potent anti-biofilm activities against staphylococci (Qin et al., 2006; Huang et al., 2012; Liu et al., 2014).

To improve the anti-bacterial activities and reduce the toxicity to mammalian cells of leading compound 5, 39 derivatives of it were designed and synthesized by introducing different functional groups, while keeping the thiazolopyrimidinone core structure intact (Zhao et al., 2014). Based on inhibitory activities against *S. epidermidis*, seven more-potent derivatives of compound 5 were screened out for further exploration of their bactericidal activity and anti-biofilm activity, as well as YycG phosphorylation inhibiting activity and cytotoxicity. The seven derivatives all displayed a strong bactericidal effect with low cytotoxicity on mammalian cells and were not hemolytic to human erythrocytes.

## Materials and methods

### Bacterial strains, culture media, and antibiotics

*S. epidermidis* ATCC 12228 (biofilm negative), *S. epidermidis* ATCC 35984 (biofilm positive), *S. aureus* ATCC 49230 and *Escherichia coli* ATCC 25922 were used in this study. Six clinical methicillin-resistant *S. aureus* (MRSA) isolates were collected from the Department of Clinical Laboratory, Shanghai General Hospital, School of Medicine, Shanghai Jiaotong University, China. Staphylococcal strains were grown in tryptic soy broth (TSB; Oxoid Ltd., Basingstoke, England), and *E. coli* strains were grown in Luria-Bertani broth medium. Mueller-Hinton broth (MHB; Oxoid Ltd., Basingstoke, England) was used for antimicrobial susceptibility testing. Vancomycin and ampicillin were obtained from the Sigma Chemical Co. (St Louis, MO, USA).

### Synthesis of the derivatives of compound 5

**Figure 1:**
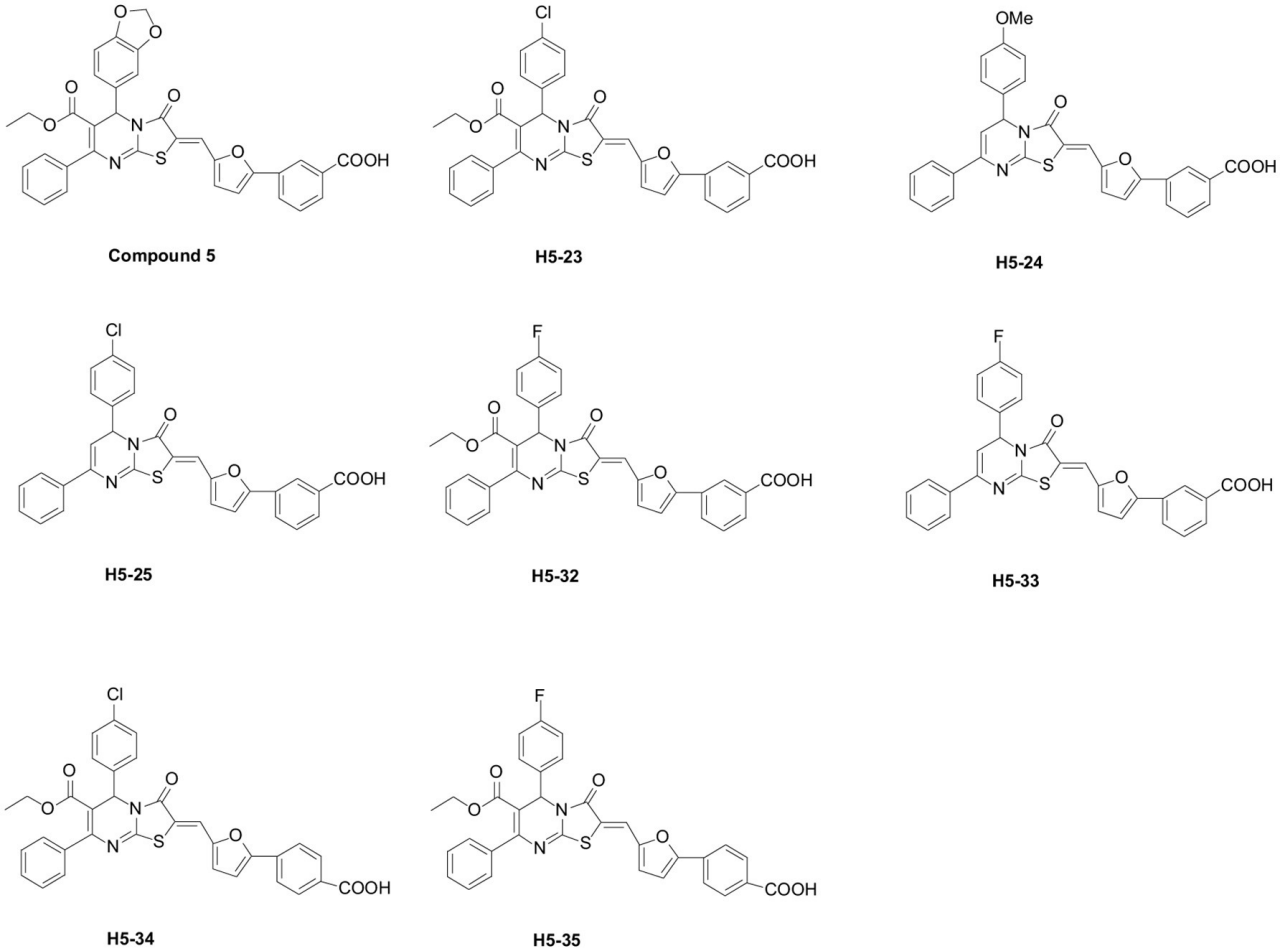
**Chemical structures of seven compound 5 derivatives**.

All the 39 derivatives of compound 5 were synthesized by introducing various chemical groups, while keeping the thiazolopyrimidinone core structure intact, and were provided by Nanjing University of Technology, China, and the Department of Natural Products Chemistry, School of Pharmacy, Fudan University, China. Based on the thiazolidione core structure of compound 5 {5-benzo [1,3] dioxol-5-yl-2-[5-(3-carboxy-phenyl)-furan-2-ylmethylene]-3-oxo-7-phenyl-2, 3 -dihydro-5H-thiazolo [3,2-a]pyrimidine-6-carboxylic} acid ethyl ester, seven derivatives were designed and synthesized by modifying the functional groups through cyclization, aldol condensation, substitution, and hydrolyzation reactions: H5-23, 2-{5-(3-carboxy-phenyl)-furan-2-ylmethylene]-5-(4-chloro-phenyl)-3-oxo-7-phenyl-2, 3-di-hydro-5H-thiazolo[3,2-a]pyrimidine-6-carboxylic acid ethyl ester; H5-24, 3-{5-[5-(4-methoxy-phenyl)-3-oxo-7-phenyl-5H-thiazolo[3,2-a]pyrimidin-2-ylidenemethyl]-furan-2-yl}-benzoic acid; H5-25, 3-{5-[5-(4-chloro-phenyl)-3-oxo-7-phenyl-5H-thiazolo [3,2-a]pyrimidin-2-ylidenemethyl]-furan-2-yl}-benzoic acid; H5-32: 2-[5-(3-carboxy-phenyl)-furan-2-ylmethylene]-5-(4-fluoro- phenyl)-3-oxo-7-phenyl-2,3-dihydro-5H-thiazolo[3,2-a]pyrimidine-6-carboxylic acid ethyl ester;H5-33, 3-{5-[5-(4-fluoro-phenyl)-3-oxo-7-phenyl-5H-thiazolo [3,2-a] pyrimidin-2-ylidenemethyl]-furan-2-yl}-benzoic acid; H5-34, 2-[5-(4-carboxy-phenyl)-furan-2-ylmethylene]-5-(4-chloro-phenyl)-3-oxo-7-phenyl-2,3-dihydro-5H-thiazolo[3,2-a]pyrimidine-6-carboxylic acid ethyl ester; H5-35, 2-[5-(4-carboxy-phenyl)-furan-2-ylmethylene]-5-(4-fluoro-phenyl)-3-oxo-7-phenyl-2,3-dihydro-5H-thiazolo[3,2-a]pyrimidine-6-carboxylic acid ethyl ester. The structural formulas of the derivatives are listed in Figure 1. Stock solutions of 200 mM derivatives were prepared in dimethylsulfoxide (DMSO; AMRESCO, USA) and stored at −80°C.

### Inhibition assay for YycG′ autophosphorylation

The construction of the recombinant plasmid pET28a-YycG′ (containing the HATPase_c and HisKA domains of YycG) has been previously described (Liu et al., 2014). After being used to transform *E. coli* strain BL21(DE3), this recombinant plasmid was induced to express YycG′ protein by 0.4 mM isopropyl-1-thio-β-D-galactopyranoside at 21°C for 12 h. Then, the bacterial cells were disrupted by sonication and centrifuged, the supernatant was purified by Ni–NTA agarose (Qiagen, Los Angeles, CA, USA). The inhibitory activities of the compound 5 derivatives on the ATPase activity of the YycG′ protein were measured using the Kinase-Glo™ Luminescent Kinase Assay (Promega, Madison, WI, USA). Briefly, 2 μg purified YycG′ protein was pre-incubated with a series of dilutions of the derivatives in reaction buffer [40 mM Tris (pH 7.5), 20 mM MgCl_2_ and 0.1 mg/ml BSA] at room temperature for 30 min, 4 μM ATP was added and incubated for 30 min at room temperature, then Kinase-Glo™ Reagent was added to detect the remaining amount of ATP, as reflected by luminescence intensity (RLU). In parallel, YycG′ protein with no addition of the derivatives was used as the control and ATP only was used as a blank. The rate of inhibition of protein phosphorylation (Rp) by the derivatives was calculated by the following equation:

$$\text{Rp​ }=\text{ ​}\frac{\begin{array}{c} \text{RLU}\left({\text{YycG}}^{\prime }+\text{derivative}+\text{ATP}+\text{Kinase}-\text{Glo}™\right)\\ -\text{RLU}\left({\text{YycG}}^{\prime }+\text{ATP}+\text{Kinase}-\text{Glo}™\right) \end{array}}{\begin{array}{c} \text{RLU}\left(\text{ATP}+\text{Kinase}-\text{Glo}™\right)\\ -\text{RLU}\left({\text{YycG}}^{\prime }+\text{ATP}+\text{Kinase}-\text{Glo}™\right) \end{array}}\text{ ​}\times \text{ ​}100$$

IC_50_ (the concentration resulting in 50% inhibition of YycG′ protein autophosphorylation) was obtained by using Origin v7.0 software (OriginLab, Northampton, USA).

Minimal Inhibitory Concentration (MIC) and Minimal Bactericidal Concentration (MBC) Assays of the Derivatives

The MIC assays of the derivatives of compound 5 against *S. epidermidis, S. aureus*, MRSA and *E. coli* were performed by using the methods described previously by Wiegand et al. (2008). In brief, two-fold serial dilutions of the derivatives were prepared in tubes containing 4 mL MHB medium, yielding final concentrations from 200 to 0.78 μM. Bacterial cultures were grown overnight and adjusted so that the bacterial suspension turbidity was equivalent to a 0.5 McFarland standard (~1 × 10^8^ CFU/mL) and further diluted to 1:200 into MHB medium. The contents in the tubes were mixed thoroughly and incubated at 37°C with shaking at 220 rpm for 16–20 h. The MIC endpoint was defined as the lowest concentration at which there was no visible growth in the tubes. The MBC values were assessed by plating 100 μL samples from each negative (no visible bacterial growth) culture tube from the MIC assays onto blank MHB agar plates. After incubation at 37°C for 24 h, the bacterial colonies on the plates were counted. The MBC was the concentration at which a 99.9% reduction of the original inoculum was observed (Pridmore et al., 2011). In this assay, all the experiments were performed three times.

### Time-killing assays of the derivatives against *S. epidermidis*

Suspensions of *S. epidermidis* (1 × 10^7^ CFU/mL) were prepared and separately treated with the derivatives of compound 5 or vancomycin at concentrations of 4 × MIC in fresh MHB. Time-kill experiments were performed at 37°C with shaking at 220 rpm under aerobic conditions. Aliquots (1 mL) were removed from the culture medium at different time points (0, 1, 3, 5, 8, and 24 h), serially diluted and 100 μL of each of the dilutions plated out onto tryptic soy agar. After incubating at 37°C for 24 h, emergent bacterial colonies were counted, and the activity of the derivatives against fast-growing *S. epidermidis* cells was determined by plotting log_10_ colony counts (CFU/mL) against time. Bactericidal activity was defined as a 3-log_10_ decrease in CFU/mL. This assay was performed in triplicate and similar results were obtained.

### Anti-biofilm activity detection of the derivatives

#### Microtiter plate assays of S. epidermidis biofilms

The biofilm-killing effect for immature (6 h old) biofilms was determined in TSB using a semi-quantitative assay. An overnight culture was diluted 1:200 into TSB medium with 0.25% glucose, and incubated in polystyrene 96-well plates at 37°C for 6 h. Biofilms were exposed to two-fold serial dilutions of the derivatives, and the plates were incubated for another 18 h at 37°C. Following the incubation and removal of non-adherent cells with 200 μl of phosphate-buffered saline, adherent biofilms were fixed with 95% methanol and stained with 2% (w/v) crystal violet. Then, the wells were washed three times with sterile distilled water and the plates were air dried for 2 h. The optical density of each well at 570 nm was determined using a 96-well plate spectrophotometer (DTX880, Beckman Coulter, USA).

#### Determination of cell viability in mature biofilms by confocal laser scanning microscope (CLSM)

The effect of the derivatives on cell viability in mature biofilms (24 h old) was determined using the Live/Dead Bacterial Viability method (Live/Dead BacLight, Molecular Probes, USA). Individual wells of cell-culture dishes (WPI, USA) were filled with diluted culture, after incubation at 37°C for 24 h, the planktonic cells were removed and fresh TSB containing the derivatives at concentrations of 4 × MIC was added and incubated at 37°C for another 16 h. The mature biofilms were stained with SYTO 9 and propidium iodide at room temperature for 15 min, following gentle washing of the dishes with normal saline, and were observed under a Leica TCS SP5 CLSM with a 63 × 1.4-NA oil-immersion objective. Further, image analysis was performed using IMARIS 7.0.0 software (Bitplane). The fluorescence quantities of biofilm were determined using Image J software. This assay was performed in triplicate and similar results were obtained.

#### Flow-based biofilm inhibition assays by bioflux

The microfluidic channels of BioFlux 48-well plates (Fluxion Biosciences, South San Francisco, CA, USA) were primed with pre-warmed (37°C) TSB medium from the outlet well at a shear setting of 2 dyn/cm^2^ for 10 min. Overnight bacterial culture of *S. epidermidis* ATCC 35984 was subcultured to mid-log phase and then diluted 1:200 in MHB medium, bacteria were seeded from the outlet well into the channel and viewing window at a shear setting of 2 dyn/cm^2^ for 3 s. After 1 h incubation for bacteria attachment at 37°C, fresh TSB medium containing 2 × MIC of the derivatives was flowed from the inlet well at a shear setting of 0.15 dyn/cm^2^. Growth of the biofilms was monitored for up to 16 h. Images of different stages were automatically acquired at 10 min intervals with bright-field illumination.

### Cytotoxicity and erythrocyte hemolysis assays

A Cell Proliferation Kit I (MTT; Roche, Indianapolis, USA) was used to detect the cytotoxicity of the derivatives against Vero cells. Vero cells were added to 96-well microtiter plates at a concentration of 5 × 10^4^ cells/well in 100 μL of culture medium. After 24 h, culture medium was replaced by 100 μL serial dilutions of the derivatives (0.78–100 μM) and the cells were incubated for 4 h in an atmosphere of 5% CO_2_, then 10 μL of the MTT stock solution was added to each well and the plate returned to the 37°C incubator for 4 h. Subsequent to the incubation, the purple formazan salts were dissolved in DMSO, and the absorbance of each well was measured at wavelengths of 595 nm (test) and 655 nm (reference). Cells treated with the solvent (0.1% DMSO) were used as a blank control. The concentration of the derivatives that produced a 50% cytotoxicity effect on Vero cells (CC_50_) was calculated using Origin v8.0 software (Origin Lab, Northampton, USA).

Hemolytic activities of the derivatives were measured on healthy human erythrocytes. A pure suspension of erythrocytes was obtained by washing a blood sample with sterile 0.9% NaCl saline solution. Erythrocytes were resuspended in normal saline to give a 5% solution, then treated with MIC, 4 × MIC or 200 μM of the derivatives for 1 h at 37°C in 96-well microtiter plates. After the incubation and centrifugation at 1,000 g for 10 min, supernatants were transferred to another sterile plate and the absorbance at 570 nm was measured on a spectrophotometric microplate reader. A positive control (100% hemolysis) and a negative control (0% hemolysis) were achieved by incubating with 1% Triton-X 100 and 0.1% DMSO, respectively. Each assay was performed in triplicate and repeated three times.

### Statistics

Experiments were performed in triplicate and repeated at least three times. One-way analysis of variance (ANOVA) was utilized for data comparison. Differences in means were considered significant when *P* < 0.05.

## Results

### Inhibition of the YycG′ protein atpase activity

To confirm the interaction of the potential YycG inhibitors with their putative target protein, recombinant YycG′ was expressed and purified (Liu et al., 2014). The enzymatic inhibitory effects of the derivatives on YycG′ kinase activity were determined by using a Kinase-Glo™ Luminescent Kinase assay. The seven derivatives displayed dose-dependent inhibition of the autophosphorylation activity of YycG′. At the concentration 100 μM, H5-23, H5-24, H5-25, H5-32, H5-33, H5-34, and H5-35 inhibited the enzymatic activity of 2 μg YycG′ protein in the presence of 4 μM ATP by 64.78, 49.92, 51.42, 55.61, 77.04, 80.72, and 63.94%, respectively. The IC_50_ values of the seven derivatives were 77.18, 82.16, 80.23, 83.91, 64.90, 61.94, and 78.19 μM, respectively (Table 1).

**Table 1:** **Biological activities of seven derivatives of compound 5**.

**Derivatives^a^**	**Molecular Formula**	**MW**	**MIC^b^(μM)**	**MBC^b^(μM)**	**MBKC^b^ (μM)**	**IC_50_^c^(μM)**	**CC_50_^d^(μM)**	**Hemolysis^e^ (%)**	
								**At MIC 200** μ**M**	
Compound 5	C_34_H_24_N_2_O_8_S	620	6.25	25	50	N/D	>200	0.31 ± 0.16	0.51 ± 0.06
H5-23	C_33_H_23_ClN_2_O_6_S	610	1.56	25	100	77.18	>200	0.27 ± 0.35	0.27 ± 0.01
H5-24	C_31_H_22_N_2_O_5_S	534	1.56	50	50	82.16	>200	0.17 ± 0.11	0.32 ± 0.05
H5-25	C_30_H_19_ClN_2_O_4_S	538	1.56	50	100	80.23	>200	0.27 ±0.01	0.39 ± 0.09
H5-32	C_33_H_23_FN_2_O_6_S	594	3.13	100	25	83.91	>200	0.15 ± 0.05	0.24 ± 0.05
H5-33	C_30_H_19_FN_2_O_4_S	522	3.13	12.5	12.5	64.90	>200	0.15 ± 0.06	0.32 ± 0.05
H5-34	C_33_H_23_ClN_2_O_6_S	610	3.13	25	25	61.94	>200	0.22 ± 0.57	0.19 ± 0.01
H5-35	C_33_H_23_FN_2_O_6_S	594	3.13	12.5	25	78.19	>200	0.17 ± 0.05	0.27 ± 0.10

*MW, molecular weight; N/D, not determined*.

a *Stock solutions (200 mM) of the derivatives were prepared in DMSO*.

b *MIC, MBC, and MBKC represent the minimal inhibitory concentration, minimal bactericidal concentration and minimal biofilm-killing concentration of the derivatives against S.epidermidis ATCC 35984*.

c *IC_50_ represents half maximal inhibitory concentration of the derivatives, which inhibit half of the autophosphorylation of recombinant YycG′ determined by the Kinase-Glo™ Luminescent Kinase Assay kit*.

d *CC_50_ represents the derivative concentration that produces 50% cytotoxicity effects on Vero cells. The highest concentration tested in the experiment was 200 μM*.

e *Hemolytic activities of the derivatives were detected on healthy human erythrocytes at their MICs and 200 μM against S epidermidis ATCC 35984*.

### Antimicrobial activity of the derivatives against planktonic bacteria

We determined the MIC values of the derivatives by using a standard tube-dilution assay. The seven derivatives (H5-23, H5-24, H5-25, H5-32, H5-33, H5-34, and H5-35) inhibited bacterial growth of *S. epidermidis* ATCC 35984, and the MIC values were 1.56, 1.56, 1.56, 3.13, 3.13, 3.13, and 3.13 μM, respectively. Furthermore, they showed similar anti-bacterial activities against *S. epidermidis* ATCC 12228 and *S. aureus* ATCC 49230, whereas none of them showed anti-bacterial activity on *E. coli* strain ATCC 25922, even at the concentration of 200 μM (Table 2). Subsequently, the bactericidal activities of these derivatives against planktonic *S. epidermidis* ATCC 35984 cells were investigated. All seven derivatives showed bactericidal activities, and the MBC values were 25, 50, 50, 100, 12.5, 25, and 12.5 μM (4 × to 32 × MIC), respectively (Table 1).

**Table 2:** **Anti-Staphylococcus activities of seven derivatives of compound 5**.

**Derivatives^a^**	**MIC^b^ (μM)**			
	***S.epidermidis* ATCC35984**	***S.epidermidis* ATCC12228**	***S.aureus* ATCC49230**	***E.coli* ATCC29522^c^**
Compound 5	6.25	6.25	6.25	>200
H5-23	1.56	1.56	1.56	>200
H5-24	1.56	1.56	1.56	>200
H5-25	1.56	1.56	1.56	>200
H5-32	3.13	6.25	3.13	>200
H5-33	3.13	3.13	3.13	>200
H5-34	3.13	3.13	3.13	>200
H5-35	3.13	3.13	6.25	>200

a *Stock solutions(200 mM) of the derivatives were prepared in DMSO*.

b *MIC of the derivatives was determined by macrodilution method of the CLSI of America*.

c *The derivatives did not inhibit the growth of E. coli ATCC 25922, even at the highest concentration used in the experiment*.

### Activities of the derivatives against fast-growing *S. epidermidis*

**Figure 2:**
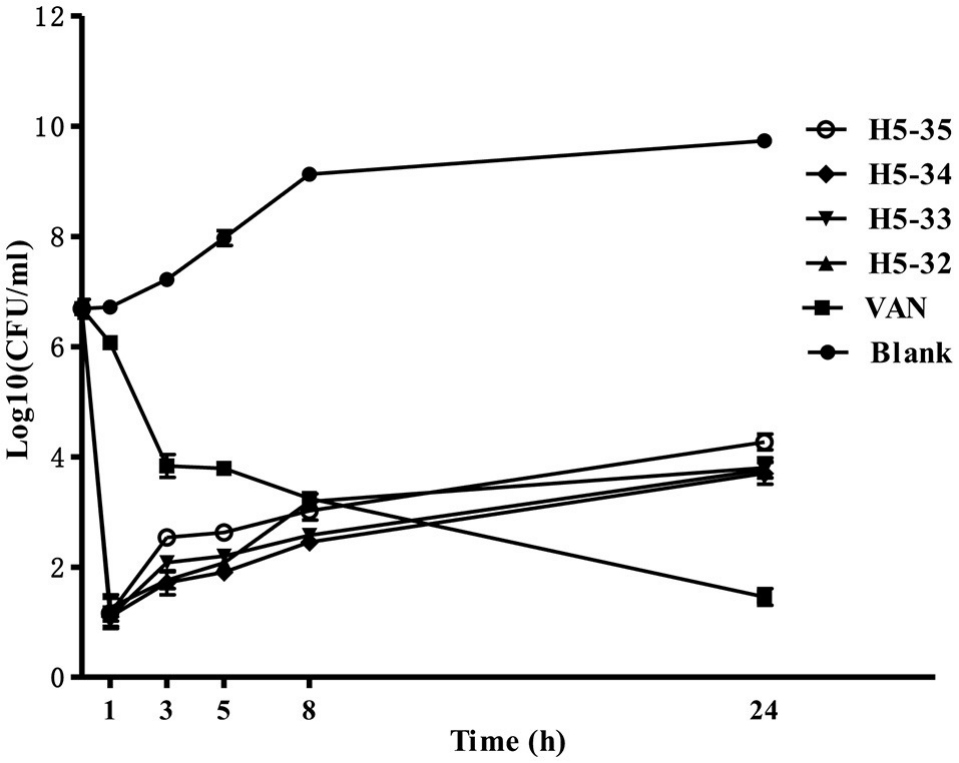
**Time-kill curves against ***S. epidermidis*** ATCC 35984**. The bacteria were separately treated with the derivatives of compound 5 or vancomycin at concentrations of 4 × MIC in fresh MHB. Aliquots (1 mL) were removed from the culture medium at different time points (0, 1, 3, 5, 8, and 24 h), serially diluted, plated out 100 μL of each of the dilutions onto tryptic soy agar. Viability was counted at the indicated time points by serial dilution plating, the activity of the derivatives against fast-growing *S. epidermidis* cells was determined by plotting log_10_ colony counts (CFU/mL) against time. Values are the mean of independent tests performed in triplicate with error bars.

We used a time-kill assay to determine the effectiveness of the derivatives against fast-growing *S. epidermidis* cells. Four derivatives (H5-32, H5-33, H5-34, and H5-35) as well as vancomycin at the concentration of 4 × MIC were separately added into fresh MHB cultures. The starting bacterial inoculum was ~1 × 10^7^ CFU/mL. Untreated control bacterial cells grew fast (increasing >3 log_10_ CFU/mL over a 24 h period). All of the derivatives exhibited bactericidal activity in growing cultures of *S. epidermidis* with a 3-log reduction in CFU/mL within 1 h, whilst vancomycin only caused a decrease of ~1 log after 1 h (*P* < 0.001), and exhibited a more protracted bactericidal activity than the four derivatives as >5 h were required to achieve a bactericidal effect (Figure 2).

### Activities of the derivatives against young biofilms

Effects of the derivatives on young biofilms of *S. epidermidis* ATCC 35984 were determined in 96-well polystyrene microtiter plates. After inoculation, the bacteria in the wells of the plates were incubated for 6 h to form immature (young) biofilms, and then two-fold serial dilutions of the derivatives were added and incubated for another 18 h. Young biofilm treated with 0.1% DMSO served as a negative control. The minimal biofilm-killing concentration ranged from 12.5 to 100 μM. The derivatives H5-32, H5-33, H5-34, and H5-35 showed stronger biofilm-killing effects at the concentrations of 12.5 or 25 μM than other derivatives (Table 1); therefore, these four derivatives were further investigated for inhibition of biofilm formation activities with a BioFlux 1,000.

**Figure 3:**
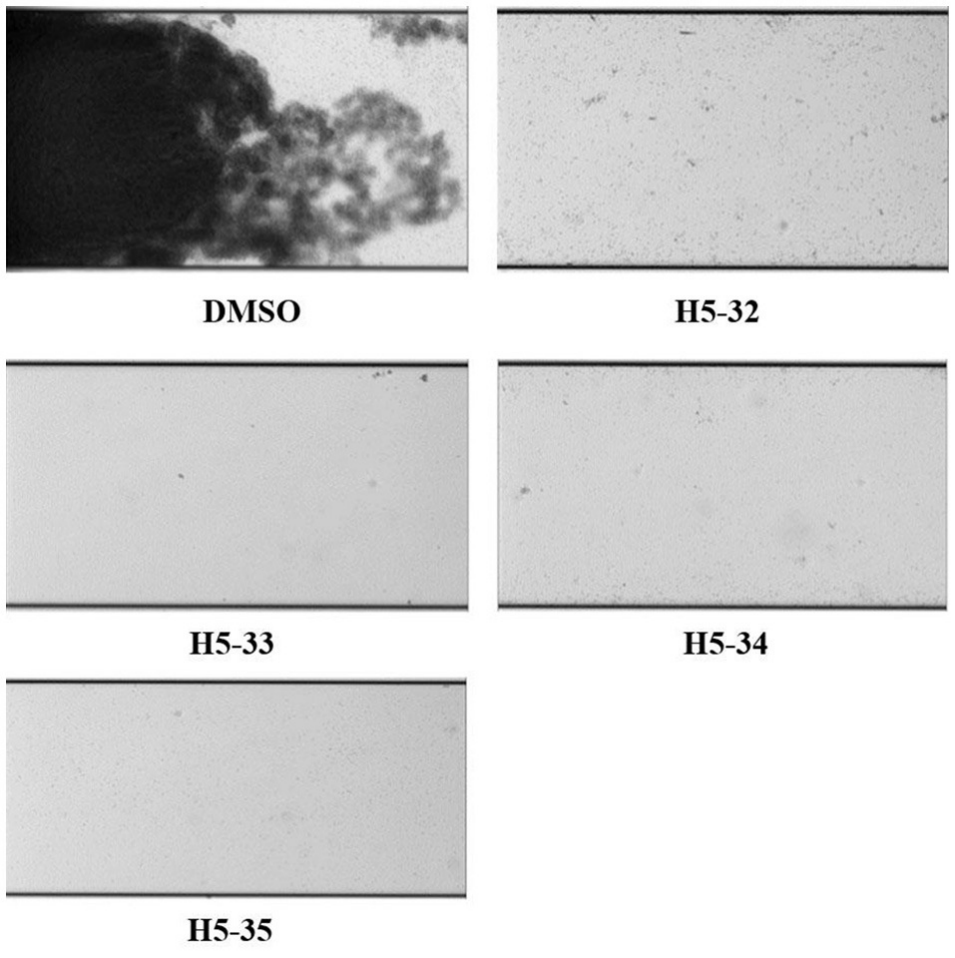
**Inhibitory effects of the derivatives on biofilm formation of ***S. epidermidis*** under flowing condition**. Overnight bacterial cultures of *S. epidermidis* ATCC 35984 were subcultured to the mid-log phase and then diluted 1:200 in MHB medium, bacteria were seeded from the outlet well into the channel and viewing window at a shear setting of 2 dyn/cm^2^ for 3 s. After 1 h incubation for bacteria attachment at 37°C, the fresh TSB medium containing 2 × MIC of the derivatives was flowed from the inlet well at a shear setting of 0.15 dyn/cm^2^. Bright-field images depicting biofilm development after 16 h were captured at 10 times the original magnification.

After pumping bacteria into the channel of the BioFlux 1,000 and incubating for 1 h, fresh TSB medium containing 2 × MIC of the derivatives was separately flowed from the inlet well at a shear setting of 0.15 dyn/cm^2^, and then biofilm formation was monitored for another 16 h. In the channels treated with the derivatives of compound 5 (H5-32, H5-33, H5-34, and H5-35) there was a significant decrease in the biofilm formation of *S. epidermidis* 35984. When grown in the absence of the inhibitors, bacteria showed initial attachment (1 h), microcolony formation (3 h), biofilm development (8 h), and firmly attached to the microfluidic flow channel and formed a robust biofilm (16 h) (Figure 3; also see the kinetic movies of biofilm formation, Supplementary Videos 1–5 in the supplemental material).

### Activity of the derivatives against mature biofilms

**Figure 4:**
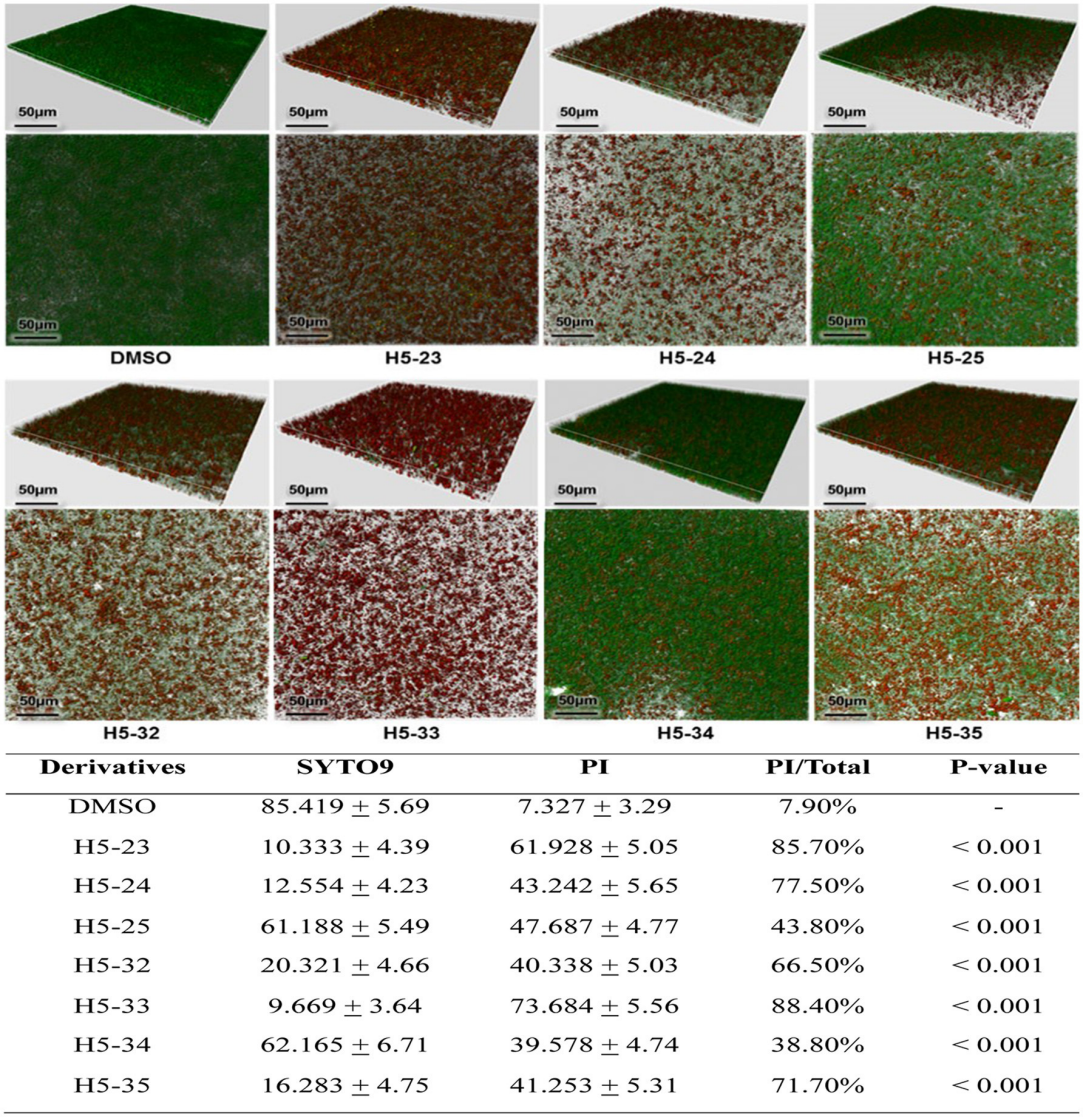
**Effects of the derivatives on mature biofilms of ***S. epidermidis*** ATCC 35984 was grown in cell-culture dishes and incubated at 37°C for 24 h, the planktonic cells were removed and fresh TSB containing the derivatives at concentrations of 4 × MIC were added and incubated at 37°C for another 16 h**. The mature biofilms were stained with SYTO 9 and propidium iodide and observed under a Leica TCS SP5 CLSM with a 63 × 1.4-NA oil-immersion objective. Images of three-dimensional biofilm structure were performed using IMARIS 7.0.0 software based on CLSM data at ~0.5 μm increments. Cells stained with green fluorescence were viable and with red fluorescence were dead. The fluorescence quantities of biofilm were determined by using Image J software. Statistical significance was determined using one-way ANOVA with respect to the control (only DMSO added). Data represents mean ± SD of three independent experiments.

The effects of the derivatives on cell viability in mature biofilms of *S. epidermidis* were detected by a CLSM with Live/Dead staining. The inoculated bacteria in the wells of plates were incubated for 24 h to form mature biofilms. All of the derivatives reduced the proportion of viable cells in mature biofilms in varying degrees, especially H5-23, H5-24, H5-33, and H5-35 (85.7, 77.5, 88.4, and 71.7%, respectively), as shown in Figure 4. As has been previously described (Liu et al., 2014), vancomycin (128 μg/mL) showed little effect on the bacterial viability in the biofilms, reduced living cells in mature biofilm only by 11.4%.

### Cytotoxicity and hemolysis of the derivatives *in vitro*

The cytotoxicity of the derivatives against Vero cells (African green monkey kidney epithelial cells) was assessed by using the MTT assay. Cells treated with 0.1% DMSO were used as a negative control. Compared with the control group, no obvious cytotoxic effects of the derivatives against Vero cells were detected, the CC_50_ values of the tested derivatives were all higher than the highest concentration used in this study (200 μM; Table 1).

All the seven derivatives displayed no obvious hemolysis on healthy human erythrocytes at MICs and even up to the concentration of 200 μM (Table 1). As a positive control, treatment with 1% Triton-X100 resulted in 100% hemolysis of human erythrocytes, while no hemolysis was observed in cells that were treated with 0.1% DMSO, as a negative control.

## Discussion

*S. epidermidis* is the main cause of biomaterial-associated infections. The most important virulence factors associated with *S. epidermidis* are related to biofilm formation, whereas *S. aureus* produces toxins and tissue-damaging exoenzymes (Vuong and Otto, 2002). Biofilms on the inert surfaces of implanted medical devices impair the penetration of antibiotics and impede the host immune response, ultimately requiring surgical removal of the infected biomaterial and subsequent replacement (Otto, 2009; Høiby et al., 2011). In this regard, effective chemical agents for inhibition and eradication of biofilms are urgently needed. With a structure-based virtual screening approach, we have found two leading compounds (compound 2 and compound 5) targeting YycG that not only were bactericidal, but also possessed potent biofilm-killing activity against *S. epidermidis* (Qin et al., 2006; Huang et al., 2012; Liu et al., 2014). Compound 2 had a thiazolidione core structure, while compound 5 had a thiazolopyrimidinone core. A series of derivatives of compound 2 have been synthesized and evaluated for their anti-bacterial and anti-biofilm activities in our previous work (Huang et al., 2012; Liu et al., 2014). In the present study, we designed and synthesized a series of derivatives of compound 5 by introducing different functional groups, while keeping the thiazolopyrimidinone core structure intact, to improve the anti-bacterial and anti-biofilm activities (Zhao et al., 2014).

The YycG/YycF (also known as WalK/WalR) TCS was first recognized in *B. subtilis* by Fabret and Fukuchi et al. and appears to be essential (Fabret and Hoch, 1998; Fukuchi et al., 2000). It is highly conserved and specific to low G+C Gram-positive bacteria, such as *B. subtilis, Streptococcus pneumoniae, S. aureus, S. epidermidis* and *Listeria monocytogenes* (Fabret and Hoch, 1998; Martin et al., 1999; Throup et al., 2000; Kallipolitis and Ingmer, 2001; Qin et al., 2006). YycG/YycF plays major roles in regulating cell-wall metabolism (Bisicchia et al., 2007; Dubrac et al., 2008), cell viability and division (Fukuchi et al., 2000; Bisicchia et al., 2007), virulence-factor expression (Ng et al., 2005; Senadheera et al., 2005), lipid integrity (Mohedano et al., 2005; Ng et al., 2005), exopolysaccharide biosynthesis and biofilm formation (Senadheera et al., 2005; Shemesh et al., 2006; Ahn and Burne, 2007). Because of its essential biological functions, the YycFG TCS has been considered as an attractive target for anti-infective therapeutics. It has been reported that inhibitors targeting WalK (YycG), walkmycin B and aranorosinol B, had anti-bacterial activity against *S. aureus* and *B. subtilis* in the low MIC range (Watanabe et al., 2003; Okada et al., 2010). Based on virtual screening, inhibitors targeting VicK (YycG) that actively inhibited the growth of *Streptococcus pneumoniae* were discovered by Li et al. (2009). Here, we mainly focused on studying the anti-biofilm properties of the newly synthesized compound 5 derivatives. By substituting different functional groups, while keeping the core structure intact, 39 derivatives of compound 5 were synthesized (Zhao et al., 2014). By testing the anti-staphylococcal activities of the derivatives, seven of them (H5-23, H5-24, H5-25, H5-32, H5-33, H5-34, and H5-35) were shown to have more potent anti-bacterial and anti-biofilm activities than leading compound 5. Additionally, four derivatives (H5-32, H5-33, H5-34, and H5-35) exhibited antimicrobial activities against six MSRA clinical isolates (MICs from 3.13 to 6.25 μM), and produced a ≥99.9% reduction in the bacterial cell count at concentrations of 8- to 32-fold higher than their MICs (see Supplementary Table 1 in the supplemental material). We also determined the antimicrobial activities of the four derivatives (H5-32, H5-33, H5-34, and H5-35) against *Enterococcus faecalis* OG1RF (from the American Type Culture Collection) by using a standard tube-dilution assay. The four derivatives displayed anti-bacterial activities on *Enterococcus faecalis* OG1RF. This was suggestive that the derivatives may be applied to infection by *Enterococcus faecalis*, although this needs further testing with more clinical strains. In the derivatives containing a substituent on the phenyl ring, such as H5-23 (MIC = 1.56 μM) and H5-25 (MIC = 1.56 μM) with a 4-Cl substituent, H5-24 with a 4-methoxy substituent (MIC = 1.56 μM) presented a much more potent anti-bacterial activity than the leading compound 5. H5-25 and H5-33, without the ester moiety on the 6 position of the core ring, showed the same anti-bacterial activity as H5-23 and H5-32, which revealed that the removal of the ester did not decrease the anti-bacterial activity. Moreover, the substitution site of the carboxyl group on the side chain may decrease the anti-bacterial activity (H5-23 vs. H5-34). Furthermore, compared with the MBCs of H5-32 and H5-33, removing the ester increased the bactericidal activity by eight times.

Bacteria within a biofilm can be 100–1,000 times more resistant to antibiotics than corresponding planktonic cells (Imaizumi et al., 2003). All the seven derivatives exhibited bactericidal activities against *S. epidermidis* cells in mature biofilms, especially the four derivatives H5-23, H5-24, H5-33, and H5-35, which significantly decreased cell viability in mature biofilms to 85.7, 77.5, 88.4, and 71.7%, respectively, at concentrations of 4 × MIC; compared to 128 μg/ml of vancomycin treatment, which only reduced *S. epidermidis* living cells in the mature biofilm by 11.4% (Liu et al., 2014). We chose one strong biofilm-forming strain of MRSA (strain 226) and tested the anti-biofilm activities of the four derivatives (H5-32, H5-33, H5-34, and H5-35) by microtiter plate assay. All derivatives showed biofilm-killing effects on 6 h young biofilms of *S. aureus*, and the minimal biofilm-killing concentration was 200 μM. The anti-biofilm activities of these YycG HK inhibitors need to be tested against more *S. aureus* strains in future studies. The seven derivatives inhibited autophosphorylation of YycG, with IC_50_ values ranging from 61.94 to 83.91 μM. This was suggestive that the derivatives may elicit their anti-bacterial and anti-biofilm effect through inhibiting the enzyme activity of the HK domain of YycG, which is an important TCS that positively regulates biofilm formation. To clarify whether the YycG inhibitors target other HKs, we cloned and expressed HisKA and HATPase_c domains of *S. epidermidis* ArlS (designated as ArlS'), which are similar to those of YycG′ (as shown in Supplementary Figure 1). The effects of the four derivatives (H5-32, H5-33, H5-34, and H5-35) on the autophosphorylation of ArlS' were determined by using a Kinase-Glo™ Luminescent Kinase Assay. None of them showed an inhibitory effect on the autophosphorylation of ArlS', even at the highest concentration (200 μM; see Supplementary Table 2 in the supplemental material). This was suggestive that the derivatives had a certain degree of specificity dependent on the characteristics of the HATPase_c domain structure of the different HKs. In addition, the derivatives showed low toxicity to mammalian cells and healthy human erythrocytes at the highest concentration (200 μM).

*S. epidermidis* is an opportunistic pathogen causing infections of implanted medical devices, primarily due to its ability to colonize and form biofilms on inert surfaces, which subsequently act as a nidus for systemic dissemination (Wang et al., 2011). The fluid dynamics of blood flow exert substantial shear forces around the indwelling devices and influence biofilm formation (Lam et al., 2014). To simulate the fluid dynamics of the *in vivo* milieu, a flow-based live cell imaging system, BioFlux 1,000, was used for developing biofilms. Four derivatives (H5-32, H5-33, H5-34, and H5-35) were selected and incubated with bacteria at a shear setting of 0.15 dyn/cm^2^ for 16 h. All of them strongly inhibited biofilm formation under conditions of shear forces, which was suggestive that the derivatives of compound 5 were suitable candidates for the prevention of biofilm formation on implanted medical devices.

In the present study, the seven newly designed derivatives with intact thiazolopyrimidinone core structures, displayed higher anti-bacterial and anti-biofilm activities than that of leading compound 5. However, two derivatives, H5-34 and H5-35, showed limited aqueous solubility characteristics at the highest concentration (200 μM) and precipitated after incubation at 37°C for 12 h, which may hinder these derivatives as drug candidates for subsequent clinical steps. The derivatives will need further modifying to improve the thiazole compounds' drug-like properties (including higher aqueous solubility and permeability, and lower toxicity), increasing their strong anti-biofilm properties for eradicating biofilm-associated infections and multidrug-resistant bacterial infections.

## Author contributions

DQ, SH, and YW designed the research; ZL, DZ, JC, and HL participated in most of the experiments; ZL, XW, JZ, RH, ZL, YS, LY analyzed the data; ZL drafted the manuscript; DQ, SH, and JC revised the manuscript.

## Funding

This work was supported by the National Natural Science Foundation of China (81271791, 81571955, 81573268, 81671982), the National High-Tech and Development Plan of China (2014AA021404), the National Science and Technology Major Project of China (2012ZX10002002), Six Talent Peaks Project in Jiangsu Province (No: 2015-SWYY-016) and the Shanghai Municipal Committee of Science and Technology (14431900300, 15431900400) and the Shanghai Municipal Committee of Science and Technology (14431900300, 15431900400).

### Conflict of interest statement

The authors declare that the research was conducted in the absence of any commercial or financial relationships that could be construed as a potential conflict of interest.
