# Supplementary material for: Anti-bacterial and Anti-biofilm Evaluation of Thiazolopyrimidinone Derivatives Targeting the Histidine Kinase YycG Protein of Staphylococcus epidermidis
Source: Front Microbiol. 2017 Mar 31;8:549. doi: 10.3389/fmicb.2017.00549 (PMC5374206; doi:10.3389/fmicb.2017.00549)
Supplement: Supplementary file 1 [file Table1.DOCX]

**Supplementary Table 1.** Anti-MRSA activities of four derivatives of compound 5

| MRSA  Strain | Sequence  Type | H5-32 | | H5-33 | | H5-34 | | H5-35 | |
| --- | --- | --- | --- | --- | --- | --- | --- | --- | --- |
|  |  | MIC | MBC | MIC | MBC | MIC | MBC | MIC | MBC |
|  |  | (μM) | | (μM) | | (μM) | | (μM) | |
| 66 | ST239 | 3.13 | 100 | 6.25 | 200 | 3.13 | 200 | 3.13 | 200 |
| 69 | ST217 | 3.13 | 50 | 6.25 | 50 | 6.25 | 50 | 6.25 | 50 |
| 141 | ST5 | 6.25 | 100 | 6.25 | 200 | 6.25 | 200 | 6.25 | 200 |
| 226 | ST59 | 6.25 | 50 | 6.25 | 200 | 6.25 | 100 | 6.25 | 100 |
| 258 | ST239 | 3.13 | 200 | 6.25 | 200 | 6.25 | 200 | 6.25 | 200 |
| 260 | ST764 | 6.25 | 100 | 6.25 | 200 | 6.25 | 200 | 6.25 | 200 |
