# Supplementary material for: Anti-bacterial and Anti-biofilm Evaluation of Thiazolopyrimidinone Derivatives Targeting the Histidine Kinase YycG Protein of Staphylococcus epidermidis
Source: Front Microbiol. 2017 Mar 31;8:549. doi: 10.3389/fmicb.2017.00549 (PMC5374206; doi:10.3389/fmicb.2017.00549)
Supplement: Supplementary file 2 [file Table2.DOCX]

**Supplementary Table 2.** Inhibiting autophosphorylation of ArlS’ protein by four derivatives of compound 5^a^

| Component | Luminescence  (RLU) | Inhibition of ArlS’ phosphorylation |
| --- | --- | --- |
| H5-32 | 17726 + 423 | -5.9% |
| H5-33 | 17786 + 382 | -5.1% |
| H5-34 | 17747 + 622 | -5.6% |
| H5-35 | 18075 + 285 | -1.5% |
| ATP | 26180 + 257 | - |
| ATP+ ArlS’ | 18196 + 207 | - |

^a^All the derivatives were used at the concentration of 200 μM, and each reaction system contained 2 μg purified protein and 4 μM ATP (see details in Materials and Methods).
