# Supplementary material for: Anti-bacterial and Anti-biofilm Evaluation of Thiazolopyrimidinone Derivatives Targeting the Histidine Kinase YycG Protein of Staphylococcus epidermidis
Source: Front Microbiol. 2017 Mar 31;8:549. doi: 10.3389/fmicb.2017.00549 (PMC5374206; doi:10.3389/fmicb.2017.00549)
Supplement: Supplementary file 8 [file Presentation1.PDF]

Supplementary Figure 1. Domain analysis of the YycG and ArlS of *S. epidermidis* RP62A. The analysis was performed based on the SMART database and the descriptions of putative functions of domains were also from SMART. HAMP: Histidine kinases, Adenylyl cyclases, Methyl binding proteins, Phosphatases domain; PAS: PER-ARNT-SIM sensor domain; HisKA: dimerization and phosphoacceptor domain; HATPase_c: ATP-binding, Phosphorylation of HisKA domain.
