# Supplementary material for: Anti-bacterial and Anti-biofilm Evaluation of Thiazolopyrimidinone Derivatives Targeting the Histidine Kinase YycG Protein of Staphylococcus epidermidis
Source: Front Microbiol. 2017 Mar 31;8:549. doi: 10.3389/fmicb.2017.00549 (PMC5374206; doi:10.3389/fmicb.2017.00549)
Supplement: Supplementary file 9 [file Presentation2.PDF]

The movie entitled as " Supplementary Video 1-5" referred as the inhibitory activities of biofilm formation of DMSO (negative control) and four derivatives (H5-32,H5-33, H5-34, H5-35), respectively under flowing condition.
