# Supplementary figures and images for: Anti-bacterial and Anti-biofilm Evaluation of Thiazolopyrimidinone Derivatives Targeting the Histidine Kinase YycG Protein of Staphylococcus epidermidis
Source: Front Microbiol. 2017 Mar 31;8:549. doi: 10.3389/fmicb.2017.00549 (PMC5374206; doi:10.3389/fmicb.2017.00549)

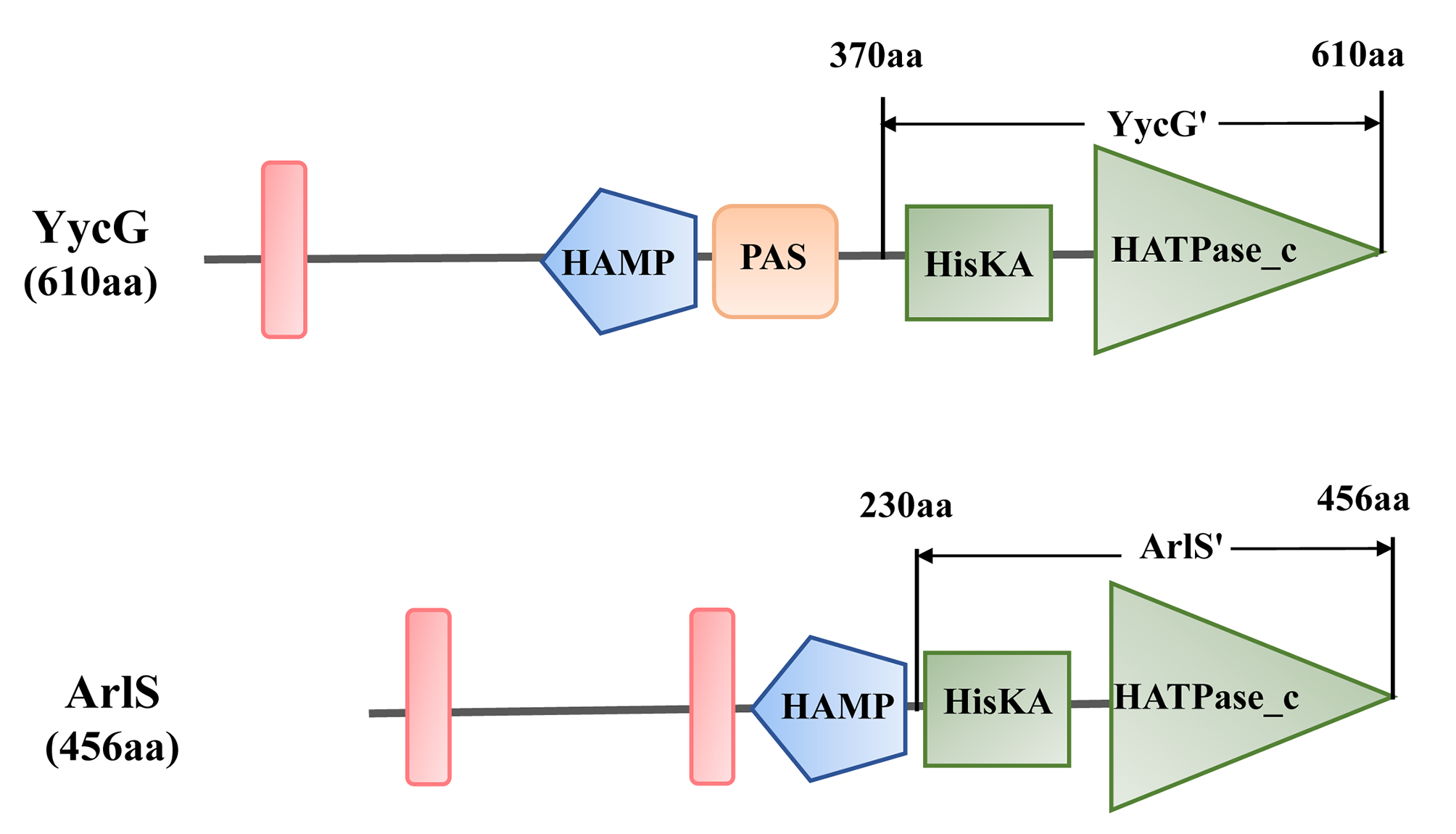

Supplement: Supplementary file 10 [file Image1.TIF]
